# Multimodal data processing for affective perception in AI-supported developmental bibliotherapy: a mixed-methods feasibility study in university libraries

**DOI:** 10.3389/fpsyg.2026.1927988

**Published:** 2026-10-06

**Authors:** Chenyang Zhou, Wei Kang

**Affiliations:** Jiangsu Second Normal University, Nanjing, China

**Keywords:** affective perception, developmental bibliotherapy, human-centered AI, multimodal data processing, student wellbeing

## Abstract

**Introduction:**

Multimodal data processing is increasingly important for understanding human affective experience in naturalistic settings. In this study, “multimodal” refers to the integration of heterogeneous human-centered evidence streams rather than synchronized perceptual or physiological sensing. We evaluated a low-threshold university-library support model integrating developmental bibliotherapy salons with AI chatbot-supported emotional expression.

**Methods:**

A convergent mixed-methods feasibility and acceptability design was conducted at a Chinese university. Of 180 baseline respondents, 45 voluntarily registered for a three-week intervention and 38 completed all sessions and post-intervention assessments; 68 non-participating students with complete baseline and follow-up assessments served as a descriptive comparison group. Evidence sources included psychological self-report data, participation records, AI-use feedback, participant evaluations, and semi-structured interviews with nine intervention participants. Quantitative indicators assessed recruitment, retention, acceptability, and exploratory SCL-90 change, while thematic analysis examined participants' affective experiences and perceived mechanisms.

**Results:**

All available intervention places were filled within two days, retention reached 84.4%, and 94.7% of completers reported moderate or considerable benefit. The intervention-group mean SCL-90 change was −0.12 points, with an approximate 95% confidence interval of [−0.23, −0.01]. Because allocation was voluntary and non-randomized, no causal group-by-time intervention effect is claimed. Qualitative findings suggested that bibliotherapy salons supported interpersonal resonance, cognitive reframing, and shared reflection, whereas the AI chatbot provided a private and non-judgmental channel for emotional disclosure.

**Discussion:**

AI-supported developmental bibliotherapy appears feasible and acceptable as a low-threshold university-library wellbeing service. The findings support feasibility, acceptability, and perceived mechanisms only; they do not establish treatment efficacy, clinical effectiveness, or a neuroscience mechanism. More broadly, the study illustrates how heterogeneous psychological, behavioral, and experiential evidence can be integrated in a naturalistic educational setting.

## 1 Introduction

University students are increasingly exposed to academic pressure, social uncertainty, career anxiety, and identity-related developmental challenges, making student wellbeing a central issue in higher education and campus service innovation (Campbell et al., 2022; Storrie et al., 2010). Psychological distress among university students has been associated with poorer academic functioning, reduced quality of life, and increased demand for campus-based support services (Bruffaerts et al., 2018). Although university counseling services remain essential, many students still encounter barriers such as stigma, limited service capacity, long waiting times, and reluctance to disclose personal distress in formal clinical settings (Castillo and Schwartz, 2013; Ebert et al., 2019). Recent studies suggest that digital mental health interventions and AI-driven conversational agents can provide scalable, low-threshold, and timely support for young people and university students, particularly when conventional services are difficult to access (Madrid-Cagigal et al., 2025). At the same time, neuroscience and perception science increasingly emphasize that emotional experience, wellbeing, and help-seeking behavior should be understood through multimodal signals, self-reported psychological states, user interaction behaviors, and contextual human experiences (Kalateh et al., 2024). This perspective creates an important opportunity to rethink university libraries not only as knowledge-service spaces, but also as accessible environments for multimodal, human-centered wellbeing support.

Existing approaches to student wellbeing have mainly followed three directions: professional counseling, digital mental health interventions, and AI-supported conversational tools. Professional counseling offers strong human support but is often constrained by limited resources and stigma-related barriers (Ebert et al., 2019). Digital interventions improve scalability and can extend support beyond traditional service hours, but they may suffer from low engagement, limited personalization, and weak contextual embedding in students' everyday campus life (Lattie et al., 2019; Organization, 2022). AI chatbots provide anonymity, accessibility, and continuous interaction, making them promising tools for emotional disclosure and low-threshold support (Feng et al., 2025). However, recent studies also report concerns about methodological heterogeneity, over-reliance on self-report, limited emotional warmth, privacy concerns, and uncertain long-term effects (Nyakhar and Wang, 2025; Reyes-Portillo et al., 2025). Evidence from university student populations further suggests that AI chatbot use should be interpreted carefully in relation to mental health outcomes, user dependence, and contextual service design (Zhang et al., 2025b). Therefore, a key research gap remains: how to design a feasible, acceptable, and ethically appropriate support model that integrates human-guided reading, AI-supported emotional expression, and mixed-methods data interpretation within a real university service environment.

To address this gap, this study proposes a multimodal AI-supported developmental bibliotherapy model situated in a university library. The proposed model combines face-to-face developmental bibliotherapy salons with AI chatbot-assisted emotional expression, thereby linking human interaction, reading-based reflection, AI-mediated dialogue, psychological self-report, and qualitative experiential data within a single feasibility framework. Rather than treating AI as a replacement for human support, the model positions AI as a supplementary channel that may lower the threshold for emotional disclosure, while bibliotherapy salons provide social resonance, shared interpretation, and guided reflection. Methodologically, this study adopts a convergent mixed-methods feasibility and acceptability design, integrating recruitment and retention indicators, participant evaluations, exploratory SCL-90 changes, AI-use feedback, and semi-structured interview data. This design responds to recent calls for application-oriented multimodal affective systems that move beyond algorithmic performance alone and examine user experience, contextual validity, and real-world implementation (Lin and Hu, 2023; Hong et al., 2024). It also aligns with recent multimodal affective computing studies that emphasize cross-modal fusion, weakly correlated data, and human-centered interpretation under complex real-world conditions (Liu et al., 2024b; Chandraumakantham et al., 2024).

A conceptual boundary is necessary. Questionnaires, participation records, self-reported AI use, evaluations, and interviews are not equivalent to synchronized perceptual or physiological modalities. They are heterogeneous data sources generated at different temporal resolutions and with different measurement assumptions. The present study therefore uses “multimodal evidence integration” as a pragmatic umbrella term, while treating “mixed-methods” and “heterogeneous evidence” as the more precise methodological descriptors. This distinction prevents the service evaluation from being confused with multimodal emotion recognition, computational sensor fusion, or neuroscience experimentation.

The main innovations and contributions of this study are summarized as follows:

- This study develops a library-based multimodal AI-supported developmental bibliotherapy model that integrates reading salons and AI chatbot interaction as complementary forms of low-threshold student wellbeing support. Unlike AI-only or reading-only interventions, the proposed service design combines a human-guided group component with a private digital reflection channel; this comparison is conceptual rather than the result of experimentally separated intervention arms.
- This study provides empirical evidence from a real university setting, showing that quantitative psychological indicators, intervention implementation records, participant evaluations, AI-use feedback, and qualitative experiential data can be jointly interpreted without overstating causal effectiveness.
- This study extends the application scope of multimodal data processing in neuroscience and perception science by demonstrating how affective, cognitive, behavioral, and contextual information can be organized within a human-centered library service model.

The remainder of this paper is organized as follows. Section 2 reviews developmental bibliotherapy, AI chatbot-based mental health support, and multimodal affective data processing. Section 3 describes the mixed-methods feasibility design, intervention procedure, measurement instruments, and data analysis strategy. Section 4 introduces the experimental environment, datasets, baselines, and evaluation protocol. Section 5 presents the empirical findings and supplementary analyses. Section 6 concludes the study and outlines future directions.

## 2 Related work

### 2.1 Developmental bibliotherapy and library-based wellbeing support

University students' psychological wellbeing has increasingly become a central concern in higher education, especially as students face academic pressure, interpersonal uncertainty, loneliness, career-related anxiety, and identity development challenges. In this context, university libraries have gradually been reconsidered as supportive campus spaces rather than merely repositories of academic resources (Ramsey and Aagard, 2018; Cox and Brewster, 2020). Their openness, familiarity, voluntary accessibility, and non-clinical atmosphere make them suitable for providing preventive and developmental wellbeing services that may reach students who are reluctant to seek formal counseling (Brewster, 2014). From the perspective of campus space theory, libraries can also function as “third places” where students experience belonging, safety, and informal social connection outside classrooms and clinical service settings.

Developmental bibliotherapy provides a theoretically relevant pathway for library-based wellbeing support. Unlike clinical bibliotherapy, which is often delivered in therapeutic contexts, developmental bibliotherapy emphasizes reading, guided reflection, emotional identification, and discussion to help individuals respond to ordinary developmental challenges. Early bibliotherapy theory suggests that reading-based activities may support self-understanding through processes such as identification with textual characters, emotional catharsis, and insight generation. Later work further highlights the role of reader advisory and structured discussion in transforming reading from a passive activity into a reflective and supportive process (Swain et al., 2014).

Recent empirical studies also indicate that reading-based interventions can contribute to emotional regulation, stress management, communication skills, and psychological wellbeing. For example, bibliotherapy has been reported to reduce examination-related stress among college students when delivered in a structured intervention format (Hamdan et al., 2021). Reading-centered interventions have also been associated with improvements in listening, communication, and reflective interpersonal understanding (Sherrill et al., 2020). More recent evidence from university library settings suggests that reading therapy may have positive effects on college students' mental health, especially when the intervention is organized through accessible institutional resources (Liu et al., 2025). Similarly, library-led bibliotherapy for stress management has shown practical feasibility in educational environments, supporting the value of librarians and campus professionals as non-clinical facilitators (Morin and LaVertu, 2025).

However, most existing studies still treat bibliotherapy as an independent reading intervention. Although these studies provide important evidence for the value of reading-based wellbeing support, they rarely examine how bibliotherapy can be combined with AI-supported emotional expression, participant evaluation, and multimodal data interpretation. In particular, little is known about how university libraries can integrate human-guided reading salons and digital interaction tools into a single low-threshold support model. This gap motivates the present study, which situates developmental bibliotherapy within a broader multimodal AI-supported framework rather than treating reading, discussion, and digital support as separate services.

### 2.2 AI chatbots and digital mental health support for students

AI-based conversational agents have become an important direction in digital mental health research because they can provide accessible, scalable, and low-pressure interaction for users who may hesitate to seek face-to-face support. A recent systematic review and meta-analysis found that AI-based conversational agents have potential for promoting mental health and wellbeing, but their effects vary according to intervention design, user engagement, target population, and evaluation method. This suggests that AI chatbots should not be evaluated only as technical systems, but also as situated support tools whose value depends on how they are embedded in real service contexts.

Recent studies further show that AI chatbots may help alleviate psychological distress and support health-related behaviors among adolescents and young adults (Feng et al., 2025). At the same time, generative AI mental health chatbots have raised new questions regarding response quality, safety, emotional appropriateness, and the boundary between supportive conversation and therapeutic intervention (Zhang et al., 2025a). These concerns are especially important in student populations, where users may engage with AI tools for emotional disclosure, self-reflection, companionship, or practical advice without clearly distinguishing between informal support and professional mental health care.

In college and university settings, recent studies have begun to examine chatbot feasibility, acceptability, and usage behavior more directly. Evidence from college student populations suggests that AI chatbots may provide a useful low-threshold support option, but existing studies remain heterogeneous in design, sample characteristics, outcome measures, and follow-up duration (Nyakhar and Wang, 2025). An open trial of a generative AI-powered mental wellness chatbot also indicates that students may accept AI-based support when it is convenient and responsive, although user trust, safety monitoring, and sustained engagement remain important concerns (Reyes-Portillo et al., 2025). In addition, research on Chinese university students suggests that AI chatbot usage behavior is associated with mental health-related outcomes, highlighting the need to interpret chatbot engagement in relation to users' psychological states, technology habits, and support needs (Zhang et al., 2025b).

Taken together, these studies indicate that AI chatbots are promising but should not be understood as substitutes for human support. AI tools can provide anonymity, immediacy, and a non-judgmental channel for emotional expression, but they may lack relational warmth, contextual sensitivity, and deep interpersonal understanding. Therefore, the present study integrates AI chatbot interaction as a supplementary channel embedded within a human-guided developmental bibliotherapy program. This design preserves the accessibility of AI-supported emotional disclosure while retaining the social resonance, shared reflection, and interpretive depth of face-to-face reading salons.

### 2.3 Multimodal data processing for affective perception and human-centered support

Multimodal data processing has become a key research direction in affective computing, neuroscience, and perception science because human emotion and wellbeing are expressed through heterogeneous channels, including language, voice, facial behavior, physiological signals, self-reports, interaction behaviors, and contextual experiences. Recent surveys on multimodal emotion recognition have summarized advances in feature representation, cross-modal fusion, robustness, and application scenarios, while also identifying persistent challenges such as modality imbalance, missing modalities, privacy concerns, and limited ecological validity (Kalateh et al., 2024). These challenges are directly relevant to real-world wellbeing-support studies, where data are often heterogeneous, partially observed, and embedded in complex social environments.

Recent multimodal affective computing studies have proposed different strategies to improve robustness under noisy, weakly correlated, or incomplete modality conditions. Transformer-based feature restoration has been used to enhance multimodal sentiment analysis when modality information is degraded or partially missing (Sun et al., 2023). Momentum distillation has also been introduced to improve multimodal learning by transferring complementary knowledge across tasks and modalities (Lin and Hu, 2023). In addition, weakly correlated multimodal sentiment analysis has highlighted the difficulty of aligning heterogeneous information sources when different modalities do not provide equally strong or consistent affective signals (Liu et al., 2024b).

Other studies have focused on modality-invariant representation, cross-modal transfer, and large-language-model-enhanced fusion. Contrastive modality-invariant learning has been proposed to reduce the negative influence of missing modalities in multimodal emotion recognition (Liu et al., 2024a). Cross-modal dynamic transfer learning further explores how information from one modality can support another when affective cues are incomplete or unevenly distributed (Hong et al., 2024). More recently, large-language-model-enhanced multimodal fusion has been investigated as a way to improve emotion interpretation by combining feature-level fusion with language-based reasoning (Chandraumakantham et al., 2024).

Uncertainty and missing-modality problems are also central to multimodal perception-oriented research. Uncertainty-aware lightweight multimodal emotion recognition has emphasized the importance of reliable prediction under ambiguous affective signals (Radoi and Cioroiu, 2024). Multimodal physiological–behavioral integration, such as the combination of EEG and facial expressions, further demonstrates the value of integrating neurophysiological and perceptual information for emotion classification (Güler and Akbulut, 2025). In addition, text-guided reconstruction methods for uncertain missing modalities show how one modality can help recover or compensate for incomplete information from another modality (Shi et al., 2025).

Despite these advances, many multimodal studies remain concentrated on algorithmic prediction, benchmark performance, and modality-level optimization. Comparatively fewer studies examine how multimodal thinking can inform human-centered wellbeing interventions in naturalistic educational environments. The present study does not aim to develop a new emotion recognition algorithm; instead, it extends the logic of multimodal data processing into a university library intervention by integrating reading-based human interaction, AI-supported emotional expression, psychological self-report, participant evaluation, and qualitative interview data. In this way, the study provides an applied perspective on how heterogeneous affective, behavioral, and experiential information can be jointly interpreted to understand student wellbeing and affective perception in a real-world support context.

For terminological precision, the manuscript distinguishes three levels of evidence: (1) conventional multimodal perceptual signals, such as speech, facial behavior, physiology, or neuroimaging; (2) digital interaction traces, such as timestamped usage logs or raw chatbot dialogue; and (3) heterogeneous service-evaluation evidence, such as questionnaires, attendance, evaluations, and interviews. The current study operates primarily at the third level and includes only self-reported summaries of the second level. Consequently, claims are limited to feasibility, acceptability, and perceived mechanisms in a naturalistic educational setting.

## 3 Method

### 3.1 Study design and multimodal feasibility framework

This study adopted a convergent mixed-methods feasibility and acceptability design to evaluate a multimodal AI-supported developmental bibliotherapy model implemented in a university library. The study was not designed as a randomized clinical efficacy trial. Instead, it aimed to examine whether the proposed model could be recruited, delivered, completed, accepted, and meaningfully experienced in a real-world educational environment. This design choice is consistent with the early-stage nature of the intervention, where the central methodological question is not whether the model produces definitive causal effects, but whether it is feasible and acceptable enough to justify more rigorous future evaluation.

#### 3.1.1 Operational definition, primary aim, and evidentiary boundary

The primary aim was to estimate implementation feasibility and participant acceptability. Exploratory symptom change was a secondary, hypothesis-generating outcome. The term “multimodal data” refers here to the coordinated interpretation of structurally different evidence sources, not to synchronized sensor channels. No latent multimodal representation was learned, no modality-level features were fused, and no prediction model was trained. The unit of integration was the study-level inference: recruitment and retention described implementation, SCL-90 scores described self-reported symptom burden, participant evaluations described acceptability, and interviews contextualized perceived mechanisms and limitations.

The corresponding hierarchy of inference was prespecified as follows. Feasibility indicators could support claims about whether the program was deliverable; acceptability indicators could support claims about perceived usefulness and burden; within-group SCL-90 change could describe temporal change among completers; and interview themes could describe participants' reported experiences. None of these components, alone or in combination, could establish that the intervention caused psychological improvement. This hierarchy guided the revised wording throughout the Results, Discussion, and Conclusion sections.

The methodological innovation of this study lies in integrating heterogeneous evidence sources into a unified feasibility-oriented framework. These sources include implementation indicators, psychological self-report, AI-use feedback, participant evaluation, and qualitative narratives. For each participant *i*, the complete multimodal evidence space is defined as


$$\begin{array}{l} X_{i}=\left\{X_{i}^{b},X_{i}^{a},X_{i}^{p},X_{i}^{q},X_{i}^{e}\right\}, \end{array}$$ (1)

where $X_{i}^{b}$ represents bibliotherapy-salon participation data, $X_{i}^{a}$ denotes AI chatbot interaction feedback, $X_{i}^{p}$ indicates psychological self-report data, $X_{i}^{q}$ represents qualitative interview data, and $X_{i}^{e}$ denotes evaluation and acceptability data. This formulation reflects the core assumption of the present study: student affective experience and wellbeing-related responses should be interpreted through multiple complementary data sources rather than a single outcome measure.

The mixed-methods structure followed a convergent design. Quantitative and qualitative data were collected during the same intervention period, analyzed separately, and integrated during the interpretation stage. This structure is expressed as


$$\begin{array}{l} D=D_{quan}⊕D_{qual}, \end{array}$$ (2)

where $D_{quan}$ denotes the quantitative dataset, $D_{qual}$ denotes the qualitative dataset, and ⊕ indicates interpretive integration rather than statistical pooling. This distinction is important because the study did not treat interview narratives as numerical labels, nor did it reduce participant experience to questionnaire scores. Instead, the two evidence streams were used to explain and complement each other.

Feasibility was conceptualized as a multidimensional construct involving recruitment, enrollment, completion, implementation stability, and acceptability. Formally, feasibility was defined as


$$\begin{array}{l} F=f\left(R,E,C,S,A\right), \end{array}$$ (3)

where *R* denotes recruitment feasibility, *E* denotes enrollment responsiveness, *C* denotes completion and retention, *S* denotes session implementation stability, and *A* denotes acceptability. This definition allowed the study to evaluate the proposed model as a service process rather than only as an outcome-generating intervention.

Exploratory psychological change was calculated using pre- and post-intervention psychological scores. For participant *i*, the individual change score was defined as


$$\begin{array}{l} \Delta P_{i}=P_{i}^{post}-P_{i}^{pre}. \end{array}$$ (4)

A negative value of Δ*P*_*i*_ indicates a reduction in self-reported psychological symptoms. However, because participants self-selected into the intervention rather than being randomly assigned, this change score was interpreted as exploratory evidence rather than causal evidence of intervention effectiveness.

To strengthen interpretive rigor, quantitative findings, participant evaluations, and qualitative themes were triangulated. For a given finding *k*, the triangulated interpretation was represented as


$$\begin{array}{l} T_{k}=g\left(Q_{k},V_{k},I_{k}\right), \end{array}$$ (5)

where *Q*_*k*_ denotes quantitative evidence, *V*_*k*_ denotes participant evaluation evidence, and *I*_*k*_ denotes qualitative interview evidence. A finding was considered more credible when these three sources converged in a theoretically coherent direction. This triangulation strategy is particularly suitable for feasibility research because implementation success, acceptability, and perceived mechanisms cannot be fully captured by a single statistical indicator.

### 3.2 Participants, recruitment, and intervention protocol

Participants were recruited through university announcements, the library's official social media account, and student online communities. These recruitment channels were selected because they were already embedded in students' everyday campus information environment and were consistent with the low-threshold positioning of the intervention. A total of 180 students completed the baseline survey. After receiving information about the intervention, 45 students voluntarily registered for the three-week program. Among them, 38 completed all three developmental bibliotherapy salons and both pre- and post-intervention assessments, forming the intervention group. In addition, 68 students who did not complete the intervention but finished both baseline and follow-up questionnaires were included as a descriptive comparison group.

Participant flow was reconstructed explicitly. Of the 180 baseline respondents, 45 registered for the intervention and 135 did not register. Among the 45 registrants, 38 completed the intervention and post-test, whereas seven did not complete the full protocol, corresponding to an intervention attrition rate of 15.6%. Among the 135 non-registrants, 68 completed the follow-up assessment and 67 did not contribute complete pre-post data. Thus, the complete-case sample of 106 participants represents 58.9% of the baseline respondents. The source package used for this revision did not contain verified individual-level reasons for the seven intervention withdrawals or the 67 non-intervention follow-up losses; no reason distribution is therefore asserted. The participant recruitment, intervention completion, and analytic flow are summarized in Figure 1. The corresponding participant counts, percentages, and analytic roles are detailed in Table 1.

**Figure 1:**
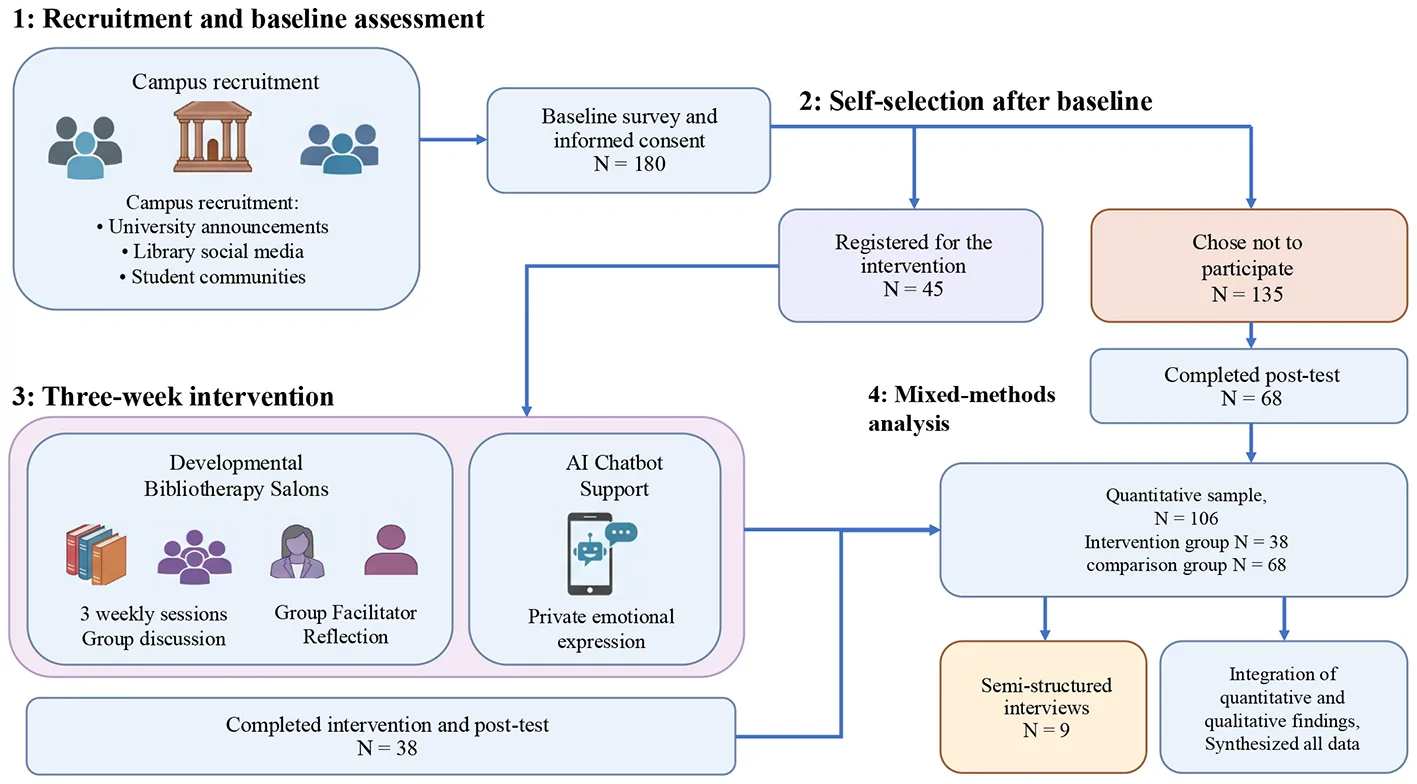
Study design and participant flow of the mixed-methods feasibility study.

**Table 1:** Reconstructed participant flow and analytic status.

Flow category	*n*	Percentage	Analytic role
Completed baseline survey	180	100.0%	Recruitment denominator.
Registered for intervention	45	25.0%	Voluntary self-selected intervention cohort.
Completed intervention and post-test	38	84.4% of registrants	Completer-only feasibility, acceptability, and exploratory pre-post analysis.
Did not complete intervention	7	15.6% of registrants	Attrition; verified reasons unavailable in the supplied source package.
Did not register for intervention	135	75.0%	Source pool for descriptive comparison follow-up.
Comparison participants with both assessments	68	50.4% of non-registrants	Baseline contextualization and descriptive comparison.
Non-registrants without complete follow-up	67	49.6% of non-registrants	Excluded from complete-case analysis.
Final complete-case sample	106	58.9% of baseline respondents	38 intervention plus 68 comparison participants.

The baseline recruitment pool was defined as


$$\begin{array}{l} N_{base}={\left\{s_{i}\right\}}_{i=1}^{180}, \end{array}$$ (6)

where *s*_*i*_ denotes the *i*-th student who completed the baseline survey. This baseline pool provided the initial population from which intervention participants and descriptive comparison participants were identified.

The final complete-case quantitative sample was composed of the intervention group and the comparison group:


$$\begin{array}{l} N_{final}=N_{int}\cup N_{comp}, \end{array}$$ (7)

where $N_{int}$ denotes the intervention group and $N_{comp}$ denotes the descriptive comparison group. The corresponding group sizes were


$$\begin{array}{l} |N_{int}|=38,\;|N_{comp}|=68,\;|N_{final}|=106. \end{array}$$ (8)

This complete-case structure ensured that both baseline and follow-up data were available for all participants included in the quantitative analysis.

Recruitment responsiveness was measured by the proportion of intervention places filled during the registration period. It was calculated as


$$\begin{array}{l} R_{cap}=\frac{N_{reg}}{N_{slot}}, \end{array}$$ (9)

where *N*_*reg*_ denotes the number of registered students and *N*_*slot*_ denotes the number of available intervention places. This indicator reflects students' initial willingness to participate in a library-based AI-supported wellbeing activity.

Retention was calculated as


$$\begin{array}{l} R_{ret}=\frac{N_{complete}}{N_{reg}}, \end{array}$$ (10)

where *N*_*complete*_ denotes the number of registered participants who completed all intervention sessions and post-intervention assessments, and *N*_*reg*_ denotes the total number of registered participants. Retention is particularly important for this study because the intervention required repeated participation across three weeks rather than one-time attendance.

Because participant-level baseline data for the seven non-completers were not included in the supplied manuscript package, a formal completer-versus-dropout comparison could not be reproduced in this revision. Attrition is therefore treated as a potential source of systematic bias rather than assumed to be random. If non-completers had greater initial distress, lower engagement, stronger privacy concerns, or less favorable experiences, the completer-only acceptability estimate could be upwardly biased. Conversely, if withdrawal was mainly logistical, bias could be smaller. The revised interpretation therefore reports retention as an implementation outcome and does not equate retention with intervention effectiveness.

The intervention consisted of three developmental bibliotherapy themes selected according to student needs identified from the baseline survey and the practical expertise of librarians and campus counselors. The theme set was defined as


$$\begin{array}{l} B=\left\{B_{1},B_{2},B_{3}\right\}, \end{array}$$ (11)

where *B*_1_ represents stress and anxiety management, *B*_2_ represents self-understanding and personal growth, and *B*_3_ represents intimate relationships and social support. These themes were designed to cover both emotional distress and broader developmental concerns.

Each participant's intervention sequence combined weekly bibliotherapy salon participation with AI chatbot-supported interaction. This sequence was represented as


$$\begin{array}{l} I_{i}={\left\{\left(B_{t},A_{t}\right)\right\}}_{t=1}^{3}, \end{array}$$ (12)

where *B*_*t*_ denotes the bibliotherapy salon in week *t*, and *A*_*t*_ denotes AI chatbot-supported interaction during the same week. This formulation emphasizes that the two intervention components were not independent add-ons, but parallel and complementary support channels embedded within the same intervention period.

Each bibliotherapy salon followed a structured process:


$$\begin{array}{l} B_{t}=\left\{r_{t},h_{t},d_{t},s_{t}\right\}, \end{array}$$ (13)

where *r*_*t*_ denotes selected reading, *h*_*t*_ denotes facilitator guidance, *d*_*t*_ denotes group discussion, and *s*_*t*_ denotes reflection and summary. The structured process was designed to support identification, emotional expression, cognitive reframing, and interpersonal resonance. Reading materials provided symbolic and narrative resources, facilitators guided interpretation, group discussion promoted social resonance, and reflection helped participants connect reading content with personal experience.

AI chatbot participation was represented as


$$\begin{array}{l} A_{i}=\left\{a_{i1},a_{i2},\ldots ,a_{im}\right\}, \end{array}$$ (14)

where *a*_*ij*_ denotes the *j*-th self-reported AI chatbot interaction of participant *i*. Because backend usage logs were unavailable due to privacy restrictions, AI engagement was treated as self-reported supportive evidence rather than objective behavioral tracking. This decision preserved participant privacy while still allowing the study to examine how students perceived and used the AI component.

To describe the conceptual relationship between the human-guided and AI-supported components, the integrated intervention exposure of participant *i* was defined as


$$\begin{array}{l} M_{i}=\alpha B_{i}+\left(1-\alpha \right)A_{i},\;0\leq \alpha \leq 1. \end{array}$$ (15)

Here, *B*_*i*_ represents bibliotherapy exposure, *A*_*i*_ represents AI-supported interaction, and α is a conceptual weighting parameter. This equation was not used to estimate a fixed statistical weight. Instead, it clarifies the methodological assumption that the intervention effect, if any, should be understood as emerging from the complementary interaction between reading-based human support and AI-assisted emotional disclosure.

### 3.3 Measures and quantitative analysis

The quantitative component focused on feasibility indicators, acceptability indicators, and exploratory psychological changes. Feasibility indicators included recruitment responsiveness, retention, session completion, and implementation stability. Acceptability indicators included satisfaction, perceived benefit, preferred themes, and participants' responses to the intervention format. These indicators were analyzed descriptively because the study aimed to evaluate feasibility and acceptability rather than establish causal clinical efficacy.

Participant satisfaction was summarized using the average satisfaction score:


$$\begin{array}{l} S_{avg}=\frac{1}{N}\overset{N}{\underset{i=1}{\sum }}S_{i}, \end{array}$$ (16)

where *S*_*i*_ denotes the satisfaction score of participant *i*, and *N* denotes the number of participants who completed the evaluation. Satisfaction was used to assess whether participants considered the intervention format acceptable and appropriate.

Perceived benefit was summarized as


$$\begin{array}{l} B_{avg}=\frac{1}{N}\overset{N}{\underset{i=1}{\sum }}B_{i}^{per}, \end{array}$$ (17)

where $B_{i}^{per}$ denotes the perceived benefit rating of participant *i*. Perceived benefit was interpreted as an acceptability-related indicator rather than a clinical outcome. This distinction prevented the study from overstating subjective evaluation as objective treatment effectiveness.

Psychological wellbeing was assessed using the Symptom Checklist-90. Let *x*_*ij*_ denote the score of participant *i* on item *j*. The total mean score was calculated as


$$\begin{array}{l} P_{i}=\frac{1}{90}\overset{90}{\underset{j=1}{\sum }}x_{ij}. \end{array}$$ (18)

This mean-score approach allowed the overall psychological symptom level to be compared before and after the intervention. Lower scores indicate fewer self-reported psychological symptoms.

The average within-group change among intervention participants was calculated as


$$\begin{array}{l} \bar{\Delta P}=\frac{1}{N_{int}}\overset{N_{int}}{\underset{i=1}{\sum }}\left(P_{i}^{post}-P_{i}^{pre}\right), \end{array}$$ (19)

where *N*_*int*_ denotes the number of intervention completers. This indicator summarizes the average direction and magnitude of pre-post change within the intervention group.

The standard deviation of the change scores was computed as


$$\begin{array}{l} SD_{\Delta }=\sqrt{\frac{1}{N_{int}-1}\overset{N_{int}}{\underset{i=1}{\sum }}{\left(\Delta P_{i}-\bar{\Delta P}\right)}^{2}}. \end{array}$$ (20)

This value reflects the variability of participants' individual changes and provides the denominator for the paired-sample test and effect size estimation.

The paired-sample statistic was calculated as


$$\begin{array}{l} t=\frac{\bar{\Delta P}}{SD_{\Delta }/\sqrt{N_{int}}}, \end{array}$$ (21)

where *t* describes whether the average within-group change differs from zero. Because the intervention was non-randomized, this statistic was used only for exploratory description.

The corresponding standardized effect size was calculated as


$$\begin{array}{l} d=\frac{\bar{\Delta P}}{SD_{\Delta }}. \end{array}$$ (22)

Cohen's *d* was used to describe the magnitude of within-group change. It was not interpreted as definitive evidence of intervention efficacy because the design did not rule out self-selection, maturation, regression to the mean, or other uncontrolled influences.

Baseline comparability between the intervention and comparison groups was examined using the standardized mean difference:


$$\begin{array}{l} SMD=\frac{{\bar{P}}_{int}^{pre}-{\bar{P}}_{comp}^{pre}}{SD_{pooled}}, \end{array}$$ (23)

where ${\bar{P}}_{int}^{pre}$ denotes the baseline mean psychological score of the intervention group, and ${\bar{P}}_{comp}^{pre}$ denotes the baseline mean psychological score of the comparison group. The pooled standard deviation was calculated as


$$\begin{array}{l} SD_{pooled}=\sqrt{\frac{\left(N_{int}-1\right)SD_{int}^{2}+\left(N_{comp}-1\right)SD_{comp}^{2}}{N_{int}+N_{comp}-2}}. \end{array}$$ (24)

These two equations were used to assess whether the two groups were similar at baseline. Any baseline imbalance was considered important when interpreting exploratory changes.

Using the reported baseline means, group sizes, and the reported between-group test, the baseline SCL-90 standardized mean difference was reconstructed as approximately 0.39, with the intervention group showing greater symptom burden. The approximate 95% confidence interval was [−0.01, 0.79]. Although the interval includes zero, the point estimate is methodologically meaningful and should not be dismissed because the corresponding *p* value was 0.059. The baseline difference is consistent with self-selection: students experiencing greater distress may have been more motivated to enroll. Therefore, regression to the mean and baseline-dependent change remain plausible alternative explanations for the observed within-group decrease.

A group-by-time model would ordinarily be preferred for comparative outcome analysis:


$$\begin{array}{l} Y_{it}=\beta_{0}+\beta_{1}Group_{i}+\beta_{2}Time_{t}+\beta_{3}\left(Group_{i}\times Time_{t}\right)+\gamma^{⊤}{\text{Z}}_{i}+\varepsilon_{it}, \end{array}$$ (25)

where β_3_ represents the differential temporal change and **Z**_*i*_ contains baseline covariates. However, the supplied source package did not include the comparison group's post-test mean, variance, or participant-level records. A valid estimate of β_3_ could therefore not be reproduced and is not reported. This omission is made explicit to prevent the within-group result from being misread as a controlled intervention effect.

The internal consistency of the SCL-90 was assessed using Cronbach's alpha:


$$\begin{array}{l} \alpha_{c}=\frac{K}{K-1}\left(1-\frac{\sum_{j=1}^{K}\sigma_{j}^{2}}{\sigma_{T}^{2}}\right). \end{array}$$ (26)

In this equation, *K* denotes the number of items, $\sigma_{j}^{2}$ denotes the variance of item *j*, and $\sigma_{T}^{2}$ denotes the variance of the total score. Cronbach's alpha was used to examine whether the psychological self-report instrument demonstrated sufficient internal consistency in the current sample.

The observed α =.974 indicates very high internal consistency, but it is not evidence of construct validity and may partly reflect item redundancy in a 90-item instrument. Accordingly, the revised manuscript does not interpret this coefficient as proof that the SCL-90 captures all relevant dimensions of student wellbeing or that the total mean score is free from common-method bias.

To summarize feasibility and acceptability in a transparent way, a descriptive feasibility-acceptability index was defined as


$$\begin{array}{l} FAI=\omega_{1}R_{cap}+\omega_{2}R_{ret}+\omega_{3}S_{avg}+\omega_{4}B_{avg}, \end{array}$$ (27)

where *R*_*cap*_ denotes recruitment capacity, *R*_*ret*_ denotes retention, *S*_*avg*_ denotes average satisfaction, and *B*_*avg*_ denotes average perceived benefit. The weights were constrained by


$$\begin{array}{l} \overset{4}{\underset{k=1}{\sum }}\omega_{k}=1. \end{array}$$ (28)

The index was used as an organizing concept rather than a definitive score. Its purpose was to make explicit how different implementation and acceptability indicators jointly informed the feasibility judgment.

The FAI was not assigned a numerical value because the component scales were not harmonized, the weights were not prospectively justified, and no validation sample was available. It is therefore retained only as a conceptual protocol template and is not used as an outcome, a hypothesis test, or evidence supporting feasibility. The empirical feasibility conclusion is based directly on separately reported recruitment, retention, and acceptability indicators. Future validation would require prespecified normalization, expert- or data-derived weights, external criteria, and sensitivity analysis across plausible weighting schemes.

### 3.4 Qualitative analysis and mixed-methods integration

The qualitative component was designed to explain how participants experienced the multimodal AI-supported developmental bibliotherapy model and why they perceived it as useful, limited, or acceptable. Nine participants from the intervention group were selected through purposive sampling to reflect diversity in gender, major, grade, intervention role, and AI-use frequency. Semi-structured interviews focused on motivations for participation, experiences of bibliotherapy salons, experiences of AI chatbot interaction, perceived changes, and suggestions for improvement.

The interview subsample was intended to maximize variation rather than statistical representativeness. The revised reporting follows COREQ principles as closely as the available records permit (Tong et al., 2007). The minimum interview guide comprised the following domains: (1) reasons for registering; (2) expectations before the first salon; (3) helpful and unhelpful aspects of reading and discussion; (4) circumstances in which the chatbot was used; (5) perceived differences between chatbot disclosure and human conversation; (6) any discomfort, privacy concern, or inappropriate response; (7) perceived changes in emotion, thinking, or behavior; and (8) suggestions for service improvement. Interviews were analyzed as experiential accounts, not as independent proof of efficacy. The interview questions and their corresponding analytic purposes are summarized in Table 2.

**Table 2:** Interview guide and analytic purpose added for qualitative transparency.

No.	Representative interview question	Analytic purpose
1	What motivated you to register for the program, and what did you expect from it?	Recruitment motivation and self-selection context.
2	Which part of the reading salon felt most or least useful?	Acceptability of the human-guided component.
3	How did group discussion affect your understanding of your own experience?	Interpersonal resonance and cognitive reframing.
4	When and why did you use the AI chatbot during the three weeks?	Context and function of AI-supported use.
5	How was talking with the chatbot different from talking with a person?	Perceived anonymity, immediacy, warmth, and limitations.
6	Did any chatbot response feel inaccurate, uncomfortable, unsafe, or overly directive?	Safety, trust, and adverse-response screening.
7	Did you notice any change in your emotions, thoughts, or daily behavior?	Participant-perceived mechanism, not causal outcome.
8	What should be changed before the service is offered again?	Feasibility refinement and implementation barriers.

The qualitative corpus was denoted as


$$\begin{array}{l} Q=\left\{q_{1},q_{2},\ldots ,q_{9}\right\}, \end{array}$$ (29)

where *q*_*i*_ represents the transcript of participant *i*. Each transcript was anonymized before analysis, and participant identifiers were replaced with pseudonyms to protect confidentiality.

The initial coding process was expressed as


$$\begin{array}{l} C=\phi \left(Q\right), \end{array}$$ (30)

where *C* denotes the set of initial codes, and ϕ(·) represents the interpretive coding process. Coding began with repeated reading of the transcripts, followed by the identification of meaningful units related to emotional expression, reading experience, AI interaction, interpersonal resonance, cognitive reframing, and perceived change.

After initial coding, codes were organized into broader themes:


$$\begin{array}{l} \Theta =\left\{\theta_{1},\theta_{2},\ldots ,\theta_{l}\right\}. \end{array}$$ (31)

Here, Θ denotes the set of final themes, and θ_*l*_ denotes the *l*-th theme. Theme development emphasized not only what participants said explicitly, but also how they described the relationship between reading, discussion, AI interaction, and perceived wellbeing-related changes.

To enhance qualitative reliability, two trained research assistants independently coded the transcripts. Intercoder agreement was assessed using Cohen's kappa:


$$\begin{array}{l} \kappa =\frac{P_{o}-P_{e}}{1-P_{e}}. \end{array}$$ (32)

In this equation, *P*_*o*_ denotes observed agreement, and *P*_*e*_ denotes agreement expected by chance. Disagreements were resolved through discussion, and the first author served as arbiter when necessary. This process helped reduce subjective coding bias and improve the trustworthiness of thematic interpretation.

The coding strategy was hybrid. Deductive sensitizing concepts were drawn from the intervention logic, including disclosure, resonance, reframing, activation, and AI limitations; inductive codes were added when participants described experiences not captured by those concepts. The codebook was iteratively revised after independent coding, and disagreements were resolved by discussion rather than by mechanically accepting the majority code. The first author documented interpretive decisions and considered how involvement in intervention design could shape theme development. Because only nine interviews were available, the study does not claim population-level saturation. Instead, the sample is described in terms of information power: the interviews were focused on a narrow intervention, but the small and self-selected sample limits thematic breadth and negative-case coverage.

No verbatim participant quotations were inserted during this revision because the uploaded source package did not contain the anonymized transcripts. Fabricating quotations would compromise qualitative trustworthiness. Representative quotations should be selected from the verified transcripts for each major theme, with pseudonymous identifiers and removal of potentially identifying details. The final audit trail should also state interview duration, setting, recording method, transcription procedure, interviewer training, prior relationships, software used, and whether participants reviewed transcripts or themes.

Mixed-methods integration was conducted after the quantitative and qualitative analyses were completed separately. The final integrated interpretation was defined as


$$\begin{array}{l} \text{Ψ}=h\left(F,\Delta P,\Theta \right), \end{array}$$ (33)

where Ψ denotes the integrated interpretation, F denotes feasibility evidence, Δ*P* denotes exploratory psychological change, and Θ denotes qualitative themes. This formulation reflects the central logic of the study: feasibility, psychological trends, and participant experience should be interpreted together.

Convergence across evidence sources was represented as


$$\begin{array}{l} C_{mix}=\frac{1}{K}\overset{K}{\underset{k=1}{\sum }}𝕀\left(Q_{k}\sim \theta_{k}\right), \end{array}$$ (34)

where 𝕀(·) is an indicator function, *Q*_*k*_ denotes the *k*-th quantitative finding, θ_*k*_ denotes the corresponding qualitative theme, and ~ indicates interpretive consistency. This equation was used conceptually to describe whether quantitative findings and qualitative themes supported the same conclusion.

Finally, the overall methodological target was summarized as


$$\begin{array}{r} M^{*}=\arg\;\underset{M}{\max}\{Feasibility(M)+Acceptability(M)\\ +ContextualValidity(M)\}. \end{array}$$ (35)

This equation captures the methodological goal of the present feasibility study. Rather than maximizing only clinical symptom reduction or algorithmic prediction accuracy, the proposed model aims to optimize feasibility, acceptability, and contextual validity in a real university library environment.

## 4 Experimental setup

### 4.1 Environment

The experiment was conducted in a real university library environment rather than in a laboratory setting. This design choice is consistent with the aim of examining whether a multimodal AI-supported developmental bibliotherapy model can be feasibly implemented and accepted as a low-threshold support service in students' everyday academic environment. The intervention was delivered over three consecutive weeks, from April 27 to May 17, 2025. Three developmental bibliotherapy salons were organized in library discussion rooms, and each session lasted approximately 75–90 min. The human-support environment involved university librarians, campus counselors, and student reading leaders. The AI-support environment was based on an AI chatbot platform that allowed text- and voice-based emotional expression, emotional journaling, and access to mental-health-related educational resources. Because the platform's privacy policy did not permit access to backend interaction logs, AI-use information was collected through participant self-report and voluntary screenshot checks.

#### 4.1.1 AI component reproducibility, safety, and privacy specification

The AI component was supportive rather than diagnostic or therapeutic. Participants could use text or voice interaction for reflection, emotional expression, journaling, and access to general educational resources. The chatbot was not treated as a substitute for professional care, and no clinical diagnosis or medication recommendation was an intended study function. The study records available for analysis did not document the platform name, provider, underlying model/version, standardized prompt, safety-filter configuration, or whether human monitoring occurred; these fields are therefore reported as not documented rather than inferred. The available and unavailable information relevant to AI-system reproducibility, safety, monitoring, privacy, and adverse-event reporting is summarized in Table 3.

**Table 3:** AI intervention reproducibility and safety reporting matrix.

Required item	Information supported by the source package	Reporting status/limitation
System type	AI chatbot platform used as a supplementary wellbeing-support tool	Verify whether commercial, institutional, or custom.
Underlying model and version	Not recorded in the supplied files	Insert verified provider, model, and version or explicitly state unknown.
Language and access mode	Text and voice interaction were available	Verify interface language, device, account type, and access period.
Prompt and conversation structure	Not recorded	State whether interaction was free-form or standardized and provide any orientation prompt.
Recommended frequency	Repeated voluntary use during the three-week program	Verify whether a minimum or recommended frequency was communicated.
Scope of advice	Reflection, expression, journaling, and educational resources	Confirm that diagnosis, treatment, and medication advice were outside scope.
Safety filters and crisis detection	Not documented in the supplied files	Describe platform safeguards and study-level screening.
Escalation pathway	Not documented in the supplied files	Insert verified referral contacts, responsible personnel, and emergency procedure.
Human monitoring	Not documented	State whether conversations were monitored and by whom.
Data processing and privacy	Backend logs unavailable; self-report and voluntary screenshots used	Verify third-party processing, voice-data handling, retention, anonymization, and deletion.
Adverse events	No verified event log was supplied	Report verified count and definition; do not infer zero from absence of documentation.

A defensible safety workflow for this type of service includes clear disclosure of AI limitations, instructions not to use the chatbot for emergencies, visible campus counseling and emergency contacts, a protocol for responding to severe SCL-90 indicators or direct reports of self-harm, and documentation of adverse or concerning chatbot responses. The authors should confirm which elements were implemented contemporaneously. Voice interaction may involve third-party processing beyond the university; the final manuscript must therefore specify data controller responsibilities, storage location, retention period, access control, and whether participants could opt for text-only use.

### 4.2 Data sources and analytic corpora

To reflect the multimodal and mixed-methods nature of the study, two datasets were constructed. In the revised terminology, these are analytic data sources or corpora rather than benchmark datasets: they were assembled for service evaluation, are not presented as publicly reusable machine-learning datasets, and contain sensitive human-subject information. Dataset 1, the Library Feasibility and Psychological Self-Report Dataset, contains complete-case quantitative records from 106 students, including 38 intervention participants and 68 descriptive comparison participants. Formally,


$$\begin{array}{l} D_{Q}={\left\{\left(x_{i},g_{i},y_{i}^{pre},y_{i}^{post},e_{i},a_{i}\right)\right\}}_{i=1}^{106}, \end{array}$$ (36)

where *x*_*i*_ represents demographic information, *g*_*i*_ group status, $y_{i}^{pre}$ and $y_{i}^{post}$ SCL-90 scores, *e*_*i*_ evaluation indicators, and *a*_*i*_ self-reported AI-use information. Dataset 2, the Interview-Based Perception Dataset, consists of semi-structured interview data from nine intervention participants:


$$\begin{array}{l} D_{I}={\left\{t_{j},m_{j},c_{j}\right\}}_{j=1}^{9}, \end{array}$$ (37)

where *t*_*j*_ denotes the anonymized transcript, *m*_*j*_ participant metadata, and *c*_*j*_ assigned codes.

### 4.3 Baselines

Since the present work is a feasibility and acceptability study rather than a randomized controlled efficacy trial, the baselines were defined as comparative research settings and analytical reference models. Table 4 summarizes the reference settings used to contextualize the proposed model.

**Table 4:** Baseline and reference settings used to contextualize the proposed model.

ID	Baseline setting	Representative study	Role in comparison
B1	Library reading-therapy baseline	University-library reading therapy for college students' mental health (Liu et al., 2025)	Represents reading-based support without AI chatbot interaction.
B2	Library-led bibliotherapy feasibility baseline	Bibliotherapy for stress management in first-year medical students (Morin and LaVertu, 2025)	Represents librarian-led bibliotherapy emphasizing stress reduction.
B3	AI conversational-agent baseline	AI conversational agents for mental health and wellbeing	Represents AI-supported psychological assistance without a library-based human reading component.
B4	Youth AI-chatbot support baseline	AI chatbots for alleviating mental distress (Feng et al., 2025)	Represents scalable chatbot support and safety evaluation.
B5	Generative AI wellness-chatbot baseline	Generative AI-powered mental wellness chatbot for college students (Reyes-Portillo et al., 2025)	Represents college-student-oriented AI wellness support.
B6	Robust multimodal affective-computing baseline	Efficient Multimodal Transformer with feature restoration (Sun et al., 2023)	Represents multimodal affective modeling under incomplete or noisy modalities.
B7	Missing-modality reconstruction baseline	Text-guided reconstruction with uncertain missing modalities (Shi et al., 2025)	Represents missing-modality reasoning relevant to unavailable backend logs.

### 4.4 Evaluation protocol

Four categories of indicators were used: implementation feasibility, participant acceptability, exploratory psychological change, and qualitative experiential interpretation. Implementation feasibility was evaluated through recruitment responsiveness, registration capacity, session completion, and retention. Acceptability was assessed through participant evaluation, perceived benefit, preferred themes, and willingness to engage with the intervention format. Exploratory psychological change was assessed using pre- and post-intervention SCL-90 scores. Qualitative analysis followed thematic analysis (Braun and Clarke, 2006; Tong et al., 2007). The final mixed-methods interpretation was based on convergence across three evidence sources:


$$\begin{array}{l} \Omega =h\left(D_{Q},D_{I},B\right), \end{array}$$ (38)

where Ω denotes the integrated interpretation, $D_{Q}$ quantitative evidence, $D_{I}$ qualitative evidence, and B baseline literature.

The comparison group was not an untreated control group created by random allocation. Its role was descriptive: to show who remained in the survey cohort without entering the program and to contextualize baseline differences. The primary analysis therefore reports feasibility and acceptability directly, and the symptom analysis is limited to a completer-based within-group estimate with a confidence interval. Covariate-adjusted causal effects, propensity-score estimates, and group-by-time effects are not presented because the necessary individual-level data and a defensible assignment mechanism were not available in the uploaded package.

## 5 Experiments and analysis

### 5.1 Overall performance comparison and analysis

The overall performance was evaluated from implementation performance and exploratory psychological outcome. The primary focus was not to claim causal clinical effectiveness, but to examine whether the proposed model could be feasibly implemented, accepted by students, and meaningfully experienced in a real university library environment.

The intervention-group mean change was $\bar{\Delta P}=1.52-1.64=-0.12$. From *t*(37) = 2.26, the standard error of the change was approximately 0.053, yielding a 95% confidence interval of approximately [−0.23, −0.01]. This interval quantifies uncertainty around the completer-only change but does not control for the comparison-group trajectory. The reported standardized within-group effect (*d* = 0.37) is small and should be interpreted as descriptive. The 94.7% perceived-benefit figure corresponds to 36 of 38 completers; the remaining two participants (5.3%) reported limited benefit. “Moderate” and “considerable” refer to the two upper response categories of the post-program perceived-benefit item, whereas “limited” denotes the lower category.

Figures 2, 3, together with Tables 5, 6, show that the proposed model achieved strong implementation performance in the university library setting. Rapid recruitment indicates that the library-based format had a low participation threshold, while the retention rate of 84.4% is encouraging for a multi-session wellbeing-support activity delivered in a non-clinical campus setting. The SCL-90 decrease from 1.64 to 1.52 provides preliminary support for a possible reduction in self-reported symptoms, although this should be interpreted cautiously because the study was non-randomized. Available baseline characteristics and group-comparability information are summarized in Table 7; unavailable summary values and covariates are reported as missing rather than inferred.

**Figure 2:**
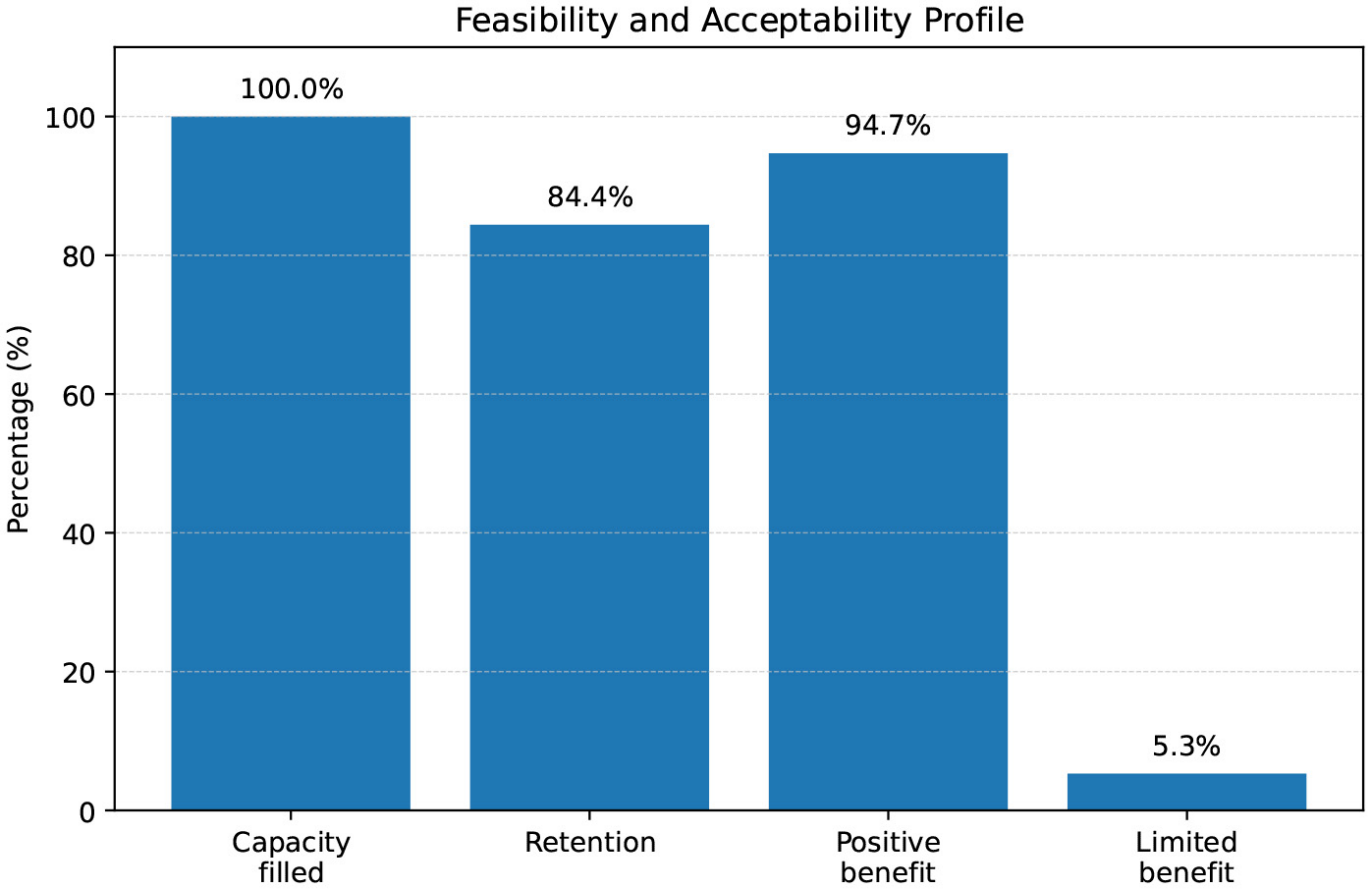
Feasibility and acceptability profile of the proposed model.

**Figure 3:**
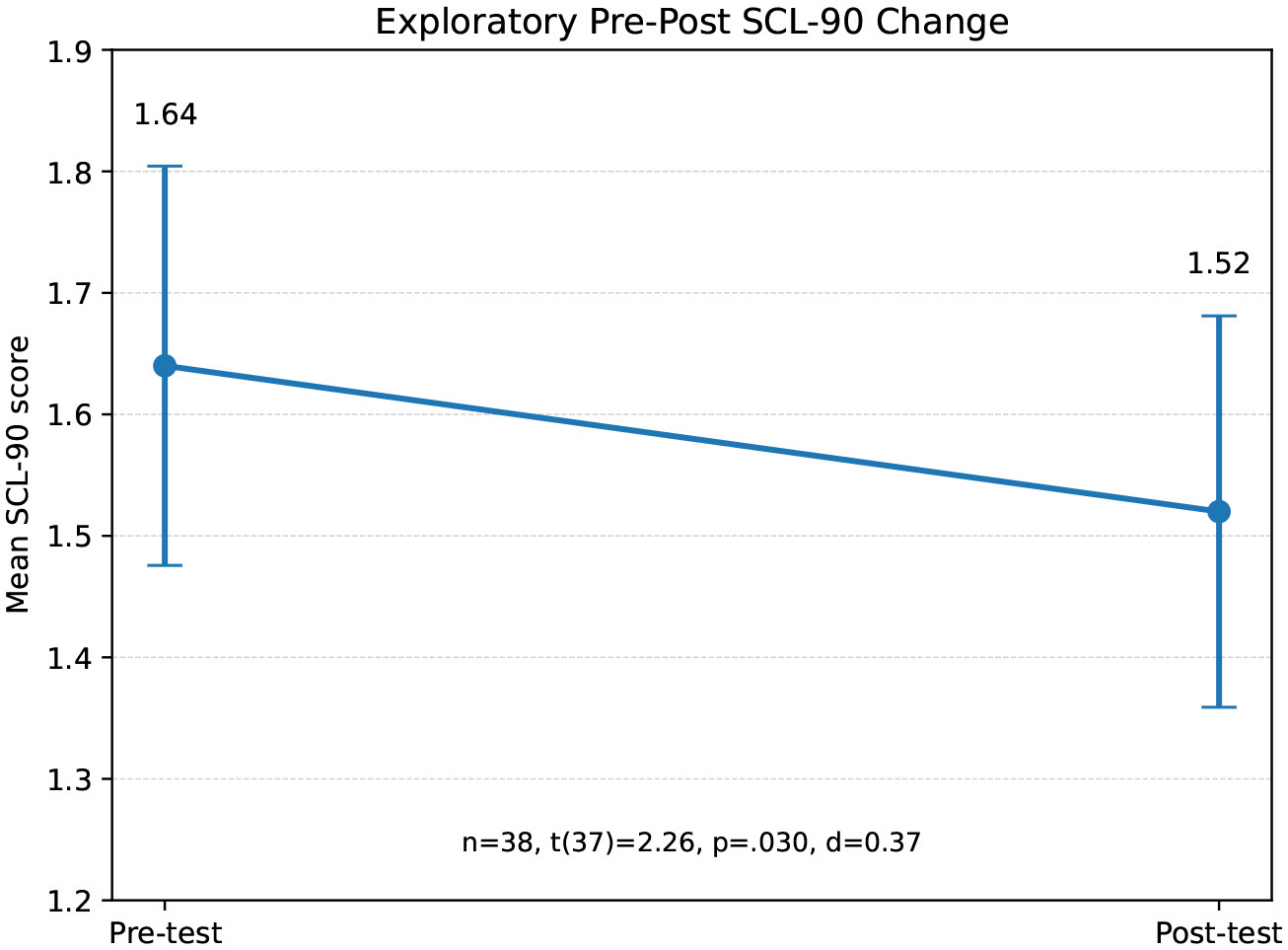
Exploratory pre-post SCL-90 change among intervention participants.

**Table 5:** Overall implementation and acceptability performance of the proposed model.

Evaluation indicator	Observed result	Interpretation
Baseline survey respondents	180 students	Broad initial recruitment base for a library-based wellbeing activity.
Available intervention places	45 places	Capacity limit of the small-group salon format.
Registration response	45/45 places	All places filled within two days, suggesting strong initial interest.
Intervention completers	38 students	Most registered participants completed all sessions and assessments.
Retention rate	84.4%	Good implementation stability for a three-week program.
Positive perceived benefit	94.7%	Most participants reported moderate or considerable benefit.
Limited perceived benefit	5.3%	Only a small proportion reported limited benefit.

**Table 6:** Exploratory psychological outcome analysis based on SCL-90 scores.

Group/comparison	*n*	Pre-test M (SD)	Post-test M (SD)	Statistic	Effect size
Proposed model	38	1.64 (0.50)	1.52 (0.49)	*t*(37) = 2.26, *p* = 0.030	*d* = 0.37
Age comparability	106	–	–	*t*(104) = −0.62, *p* = 0.54	–
Gender comparability	106	–	–	χ^2^(1, *N* = 106) = 0.39, *p* = 0.53	–
Baseline SCL-90 difference	106	Intervention: 1.64; comparison: 1.48	–	*p* =.059	Marginal imbalance

**Table 7:** Baseline characteristics and group-comparability information available in the supplied source package.

Characteristic	Intervention (*n* = 38)	Comparison (*n* = 68)	Comparison metric	Interpretation/verification status
Age	Summary values not supplied	Summary values not supplied	*t*(104) = −0.62, *p* = 0.54	No evidence of a mean-age difference from the reported test; means and SDs from original data.
Gender	Counts not supplied	Counts not supplied	χ^2^(1, *N* = 106) = 0.39, *p* = 0.53	No evidence of a distributional difference from the reported test; category counts.
Academic year	Not supplied	Not supplied	Not reproducible	
Discipline / major	Not supplied	Not supplied	Not reproducible	
Previous mental-health service use	Not supplied	Not supplied	Not reproducible	Important potential confounder.
Prior AI-chatbot use	Not supplied	Not supplied	Not reproducible	Important technology-exposure covariate.
Baseline SCL-90 mean (SD)	1.64 (0.50)	1.48 (SD not supplied)	*p* = 0.059; approximate SMD = 0.39	Moderate point-estimate imbalance consistent with self-selection; approximate 95% CI for SMD [−0.01, 0.79].

### 5.2 Conceptual component-level mechanism analysis

Because the intervention was not randomized into separate component groups, the ablation analysis was conducted at the design and mechanism level. It evaluates what type of evidence or support function would be weakened if one component were removed.

This section is not an empirical ablation study. Participants were not assigned to salon-only, chatbot-only, library-free, or mixed-methods-free variants. The table therefore summarizes hypothesized component functions and participant-perceived mechanisms; it does not estimate component effects. The resulting conceptual component-level mechanism analysis is summarized in Table 8; it should be interpreted as a design-level comparison rather than an empirical ablation or dismantling experiment. Statements about the “full model” are interpreted as design propositions requiring future factorial, dismantling, or multi-arm evaluation.

**Table 8:** Conceptual component-level mechanism analysis of the proposed model; no experimental dismantling was conducted.

Model variant	Removed component	Expected weakened function	Observed evidence supporting the full model
Full proposed model	None	Integrated support through reading, group reflection, AI expression, and mixed-methods evaluation.	High recruitment, 84.4% retention, 94.7% positive benefit, and exploratory SCL-90 reduction.
Without salons	Human-guided reading and group discussion	Reduced interpersonal resonance and meaning-making.	Interviews showed that salons promoted belonging and perspective-taking.
Without AI chatbot	Private AI-supported emotional expression	Reduced immediate, private, and low-pressure disclosure.	Participants described the chatbot as a private space for feelings difficult to share with others.
Without library context	Low-threshold campus environment	Reduced accessibility and non-clinical atmosphere.	Rapid recruitment suggests that the library context lowered barriers.
Without mixed methods	Qualitative or quantitative evidence source	Reduced ability to explain why the model was acceptable.	Quantitative indicators demonstrated feasibility, while interviews explained perceived mechanisms.

The integrated design was intended to provide a broad support structure by combining human-guided developmental bibliotherapy, AI-supported emotional expression, and mixed-methods evaluation; however, the present data cannot demonstrate that it is superior to any isolated component. Compared with AI-only tools, the proposed model preserves human interaction and group reflection. Compared with conventional reading therapy, it adds an AI-supported channel that extends emotional expression beyond the face-to-face session.

### 5.3 Participant attrition, selection bias, and sensitivity of interpretation

Seven of 45 registrants did not complete the full intervention, and 67 of 135 non-registrants did not provide complete follow-up data. Because verified withdrawal reasons and individual baseline profiles were not supplied, missingness cannot be assumed to be completely at random. The complete-case analysis may overrepresent participants with greater availability, stronger engagement, more favorable experiences, or fewer privacy concerns. The high perceived-benefit proportion should therefore be understood as the proportion among completers, not among all registrants or all baseline respondents.

The voluntary allocation mechanism also creates confounding by indication: students with higher baseline distress may have been more likely to seek the intervention. The baseline SCL-90 point estimate supports this possibility. Regression to the mean could then produce a pre-post reduction even without an intervention-specific effect. A future confirmatory study should prespecify a primary outcome, use randomized or matched allocation, retain reasons for non-participation and withdrawal, conduct intention-to-treat analysis, use multiple imputation or inverse-probability weighting for missing outcomes, and report a group-by-time estimate with covariate adjustment.

### 5.4 Mechanism-oriented and mixed-methods analysis

Figure 4 and Table 9 show that the two intervention components served distinct but complementary functions. AI mainly supported private emotional disclosure and low-pressure interaction, while the bibliotherapy salons supported interpersonal resonance and cognitive reframing. This two-layer structure explains why the integrated model is more suitable than a single-channel intervention.

**Figure 4:**
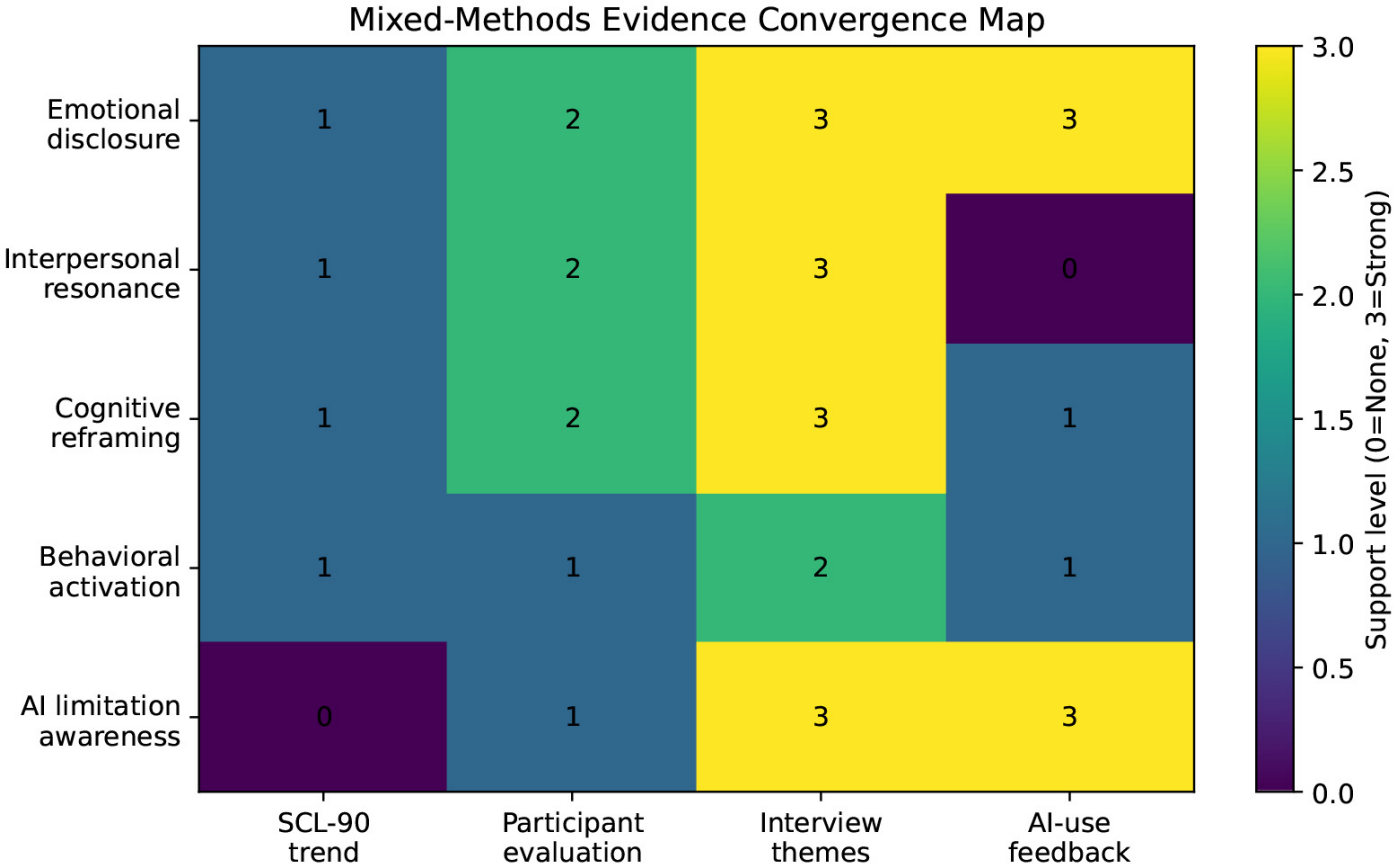
Mixed-methods evidence convergence map.

**Table 9:** Mechanism-oriented analysis of the proposed model.

Mechanism	Primary supporting component	Observed participant experience	Role in the full model
Emotional disclosure	AI chatbot support; group sharing	AI provided a private and non-judgmental space for expression.	Lowers the threshold for expressing distress.
Interpersonal resonance	Developmental bibliotherapy salons	Participants recognized that others had similar concerns.	Strengthens belonging and social support.
Cognitive reframing	Reading materials and facilitator guidance	Participants reconsidered responsibility, self-blame, and alternative interpretations.	Supports reflective thinking and perspective-taking.
Behavioral activation	Reflection and post-session practice	Some participants reported more adaptive self-talk and emotional regulation.	Converts insight into daily behavioral adjustment.
AI limitation awareness	AI chatbot interaction	Participants valued AI but preferred human comfort in intense emotional situations.	Clarifies that AI should supplement rather than replace human support.

### 5.5 Supplementary experimental results

Figures 5–7 summarize the supplementary descriptive results on role-specific AI engagement, bibliotherapy-theme preferences, and perceived benefit after the intervention. The role-AI engagement matrix shows that AI chatbot engagement was not restricted to a small subset of highly active users; both general participants and reading leaders reported repeated use during the intervention period. The theme-preference distribution indicates that self-understanding and stress reduction were equally important, suggesting that students participated not only because of distress but also because of broader developmental needs. The perceived-benefit distribution further supports the acceptability of the proposed model, with 36 of 38 completers reporting moderate or considerable benefit.

**Figure 5:**
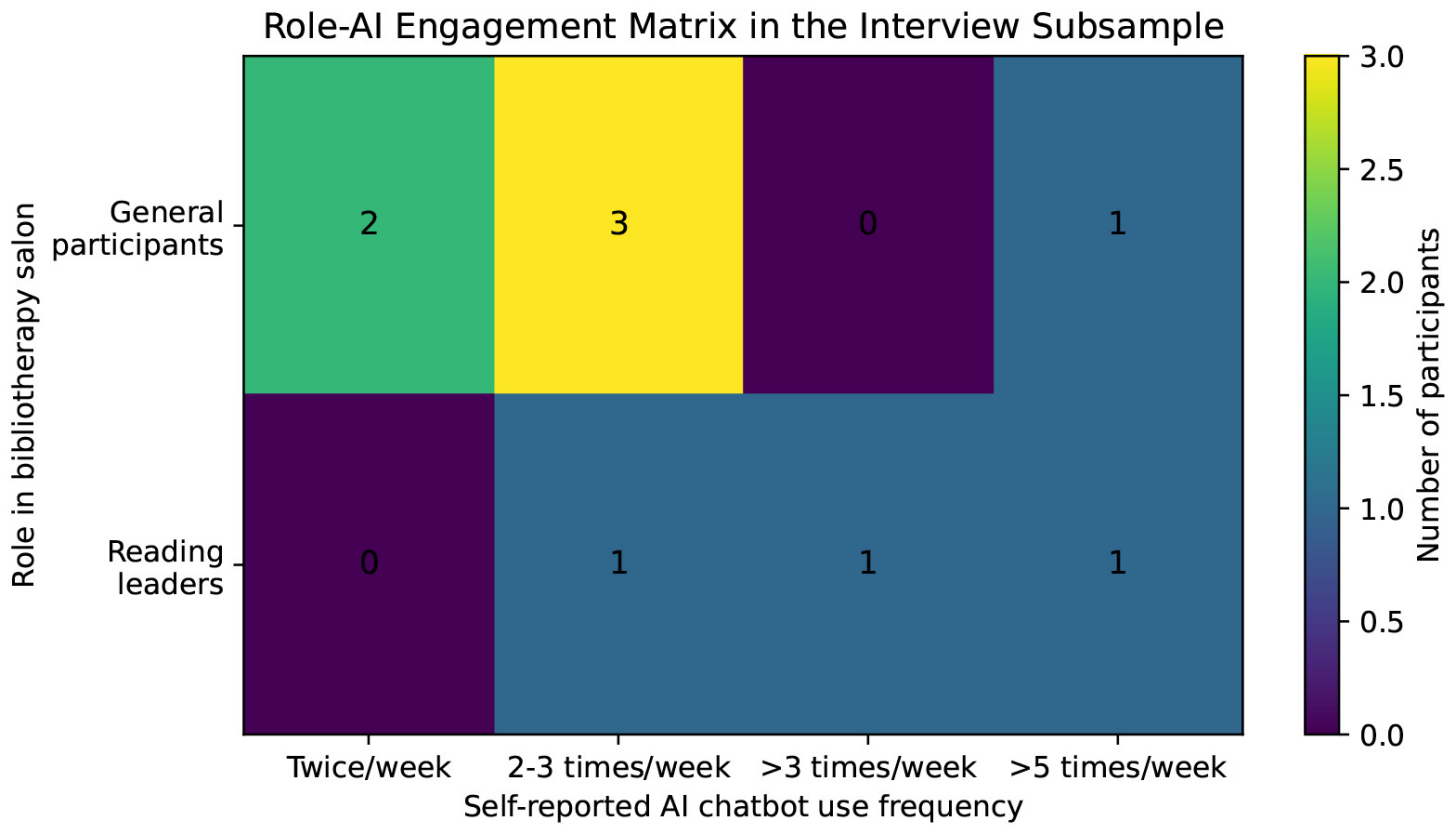
Role-AI engagement matrix in the interview subsample.

**Figure 6:**
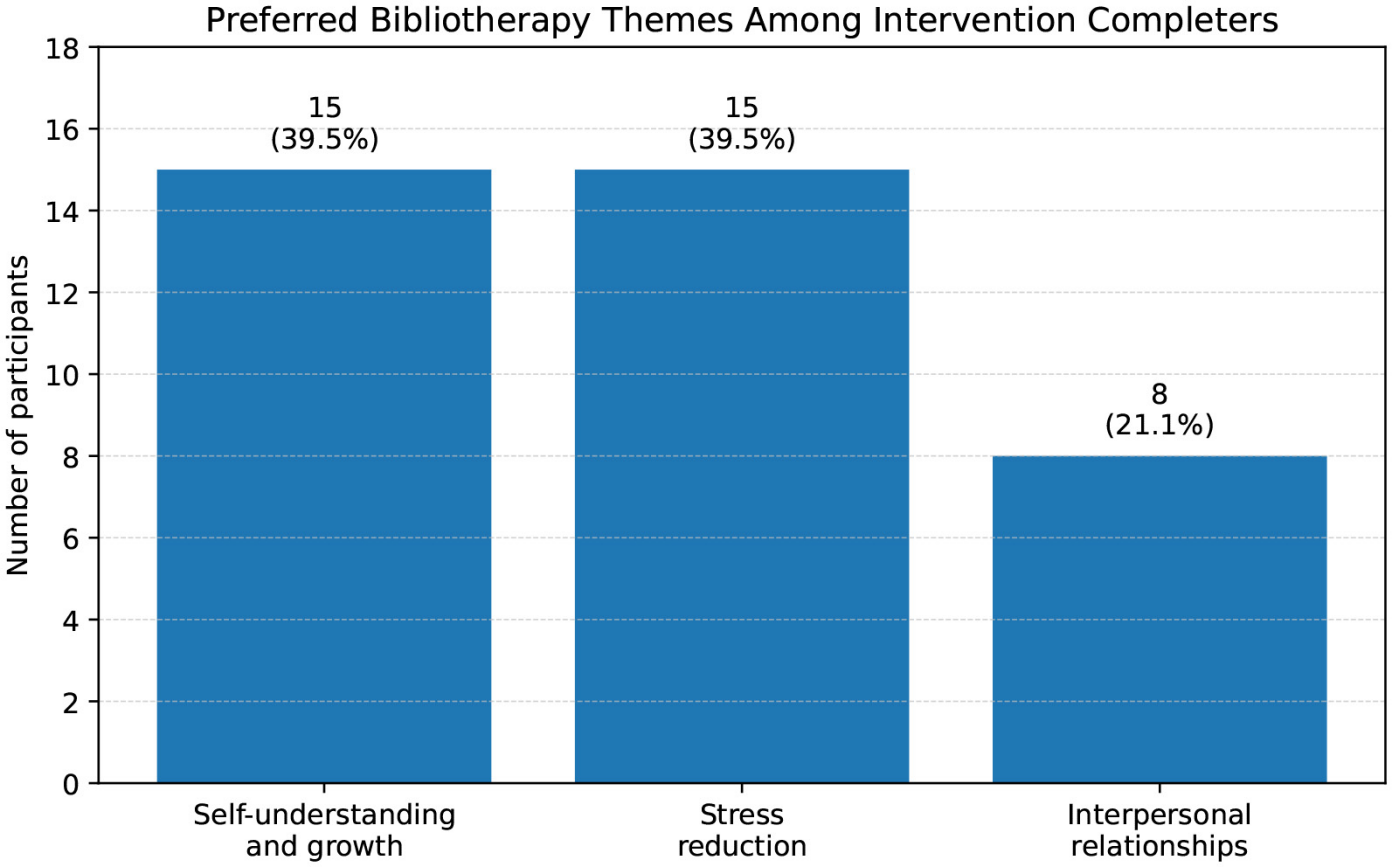
Preferred bibliotherapy themes among intervention completers.

**Figure 7:**
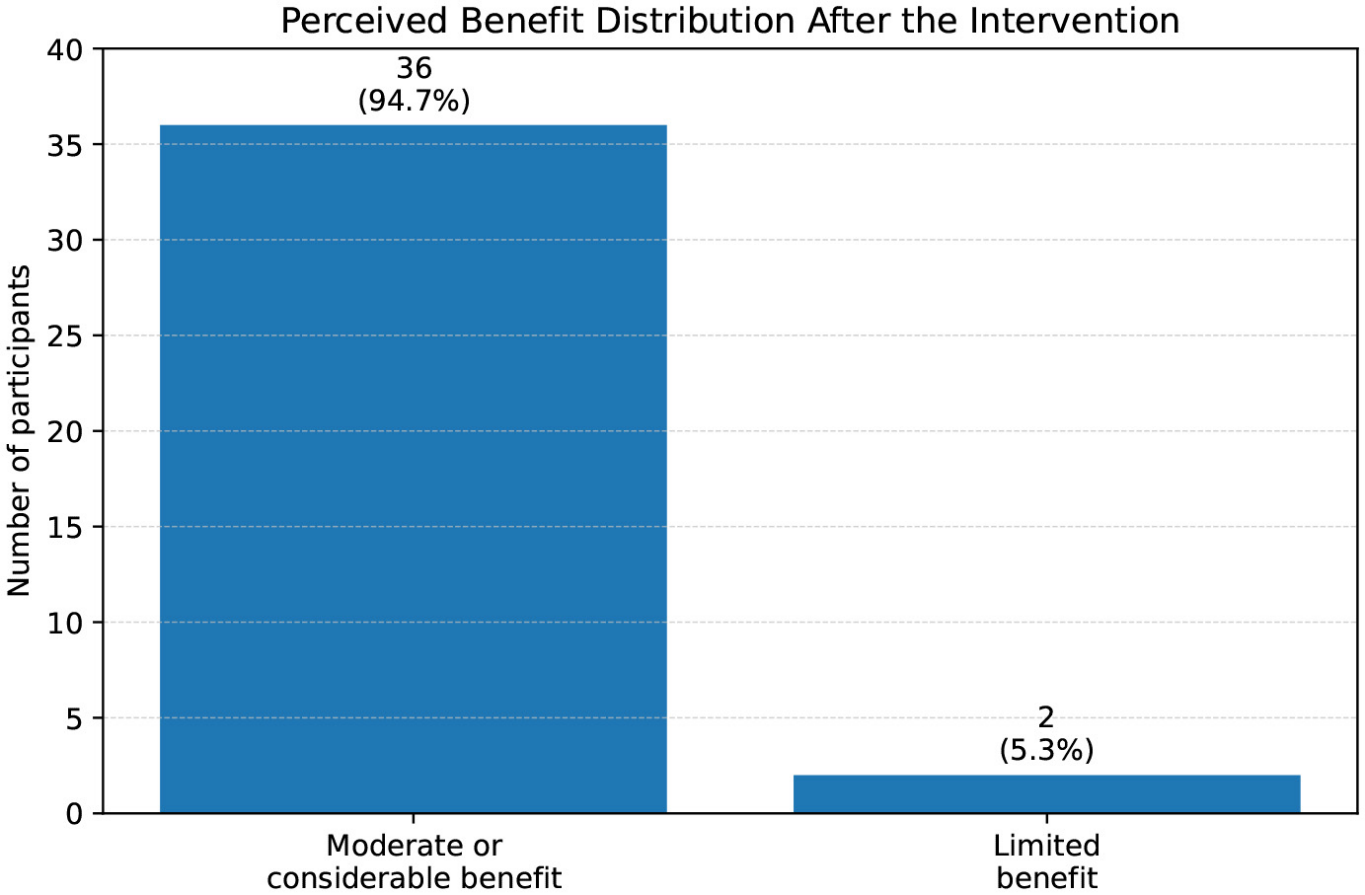
Perceived benefit distribution after the intervention.

### 5.6 Robustness, error analysis, and clustering visualization

Robustness was evaluated from implementation stability, measurement reliability, baseline comparability, qualitative coding consistency, and AI-use uncertainty. Table 10 summarizes the evidence. Figures 8–11 provide the exploratory PCA, hierarchical clustering, normalized centroid profiles, and participant-feature heatmap used only to visualize case-level heterogeneity in the nine-participant interview subsample.

**Table 10:** Robustness and sensitivity analysis of the mixed-methods evidence.

Dimension	Observed result	Potential risk	Interpretation
Implementation stability	Retention = 84.4%	Dropout across repeated sessions	Acceptable stability across three weeks.
Initial recruitment	45/45 places in two days	Low willingness to participate	Rapid recruitment suggests accessibility.
Internal consistency	Cronbach's α =.974	Unreliable measurement	The SCL-90 showed excellent internal consistency.
Age comparability	*t*(104) = −0.62, *p* =.54	Demographic imbalance	No significant age difference.
Gender comparability	χ^2^(1, *N* = 106) = 0.39, *p* =.53	Gender imbalance	No significant gender difference.
Baseline imbalance	1.64 vs. 1.48, *p* =.059	Self-selection bias	Students with higher initial distress may have been more likely to participate.
Qualitative coding	κ =.964, *p* < .001	Subjective coding bias	High intercoder agreement supports thematic reliability.
AI-use verification	Self-report and screenshots	Lack of backend logs	AI-use evidence should be interpreted cautiously.

**Figure 8:**
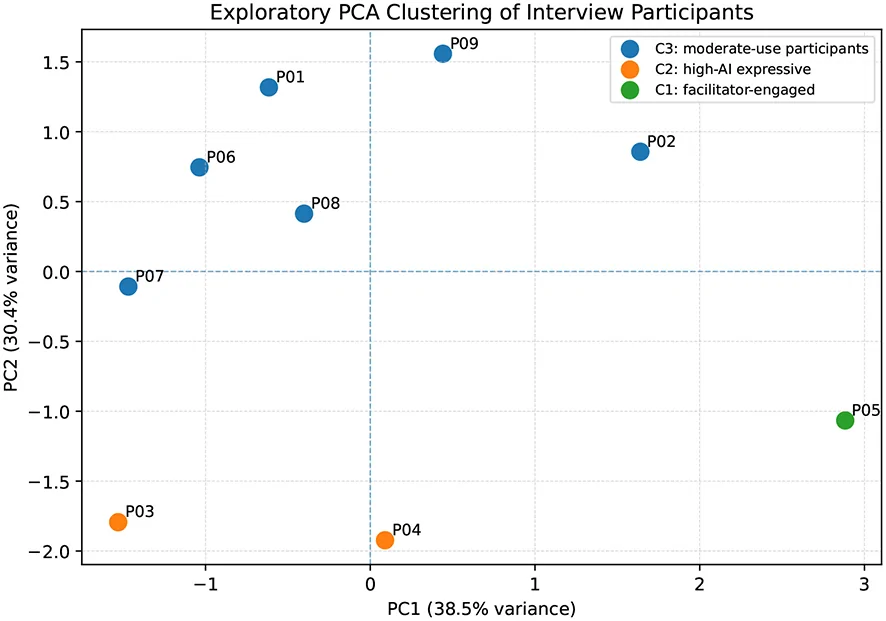
Purely exploratory PCA visualization of nine interview participants; not a validated clustering result.

**Figure 9:**
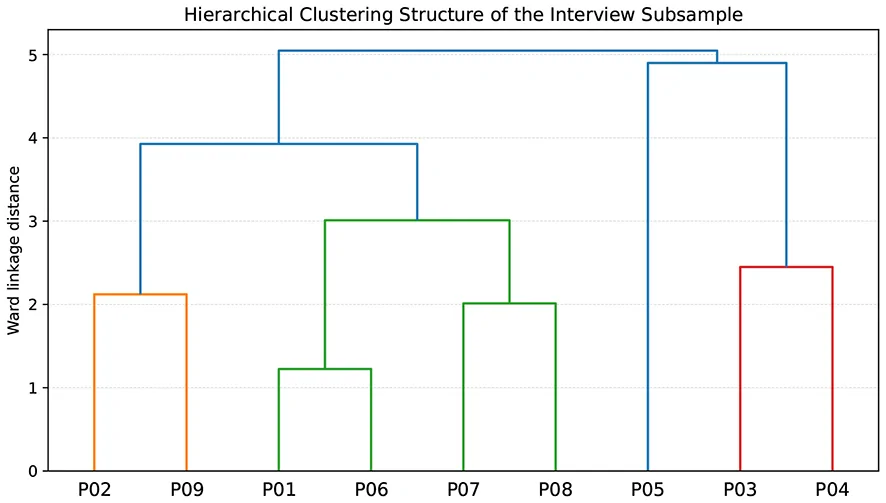
Purely exploratory hierarchical visualization of the nine-participant interview subsample.

**Figure 10:**
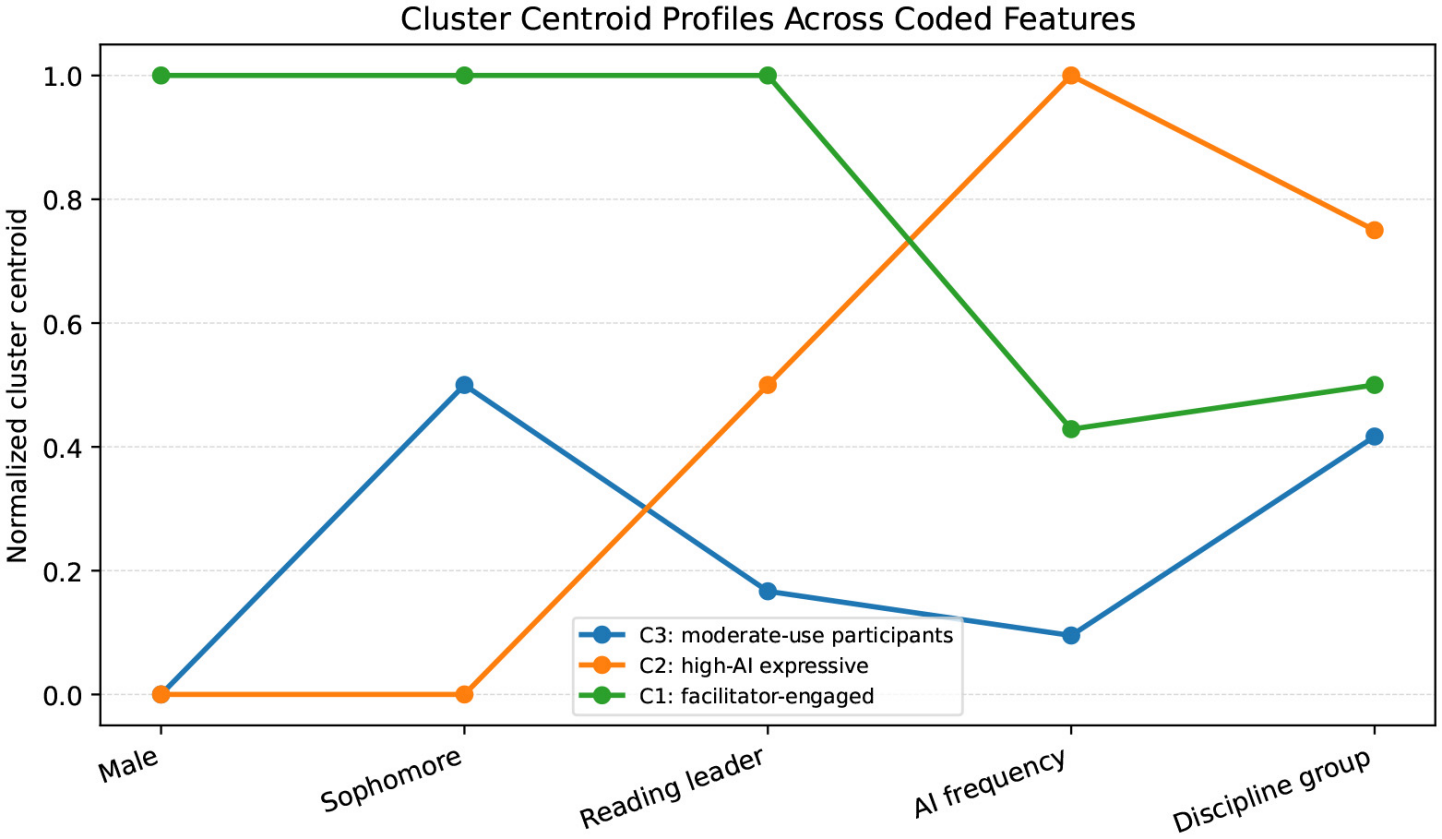
Descriptive centroid profiles generated from the nine-person exploratory visualization.

**Figure 11:**
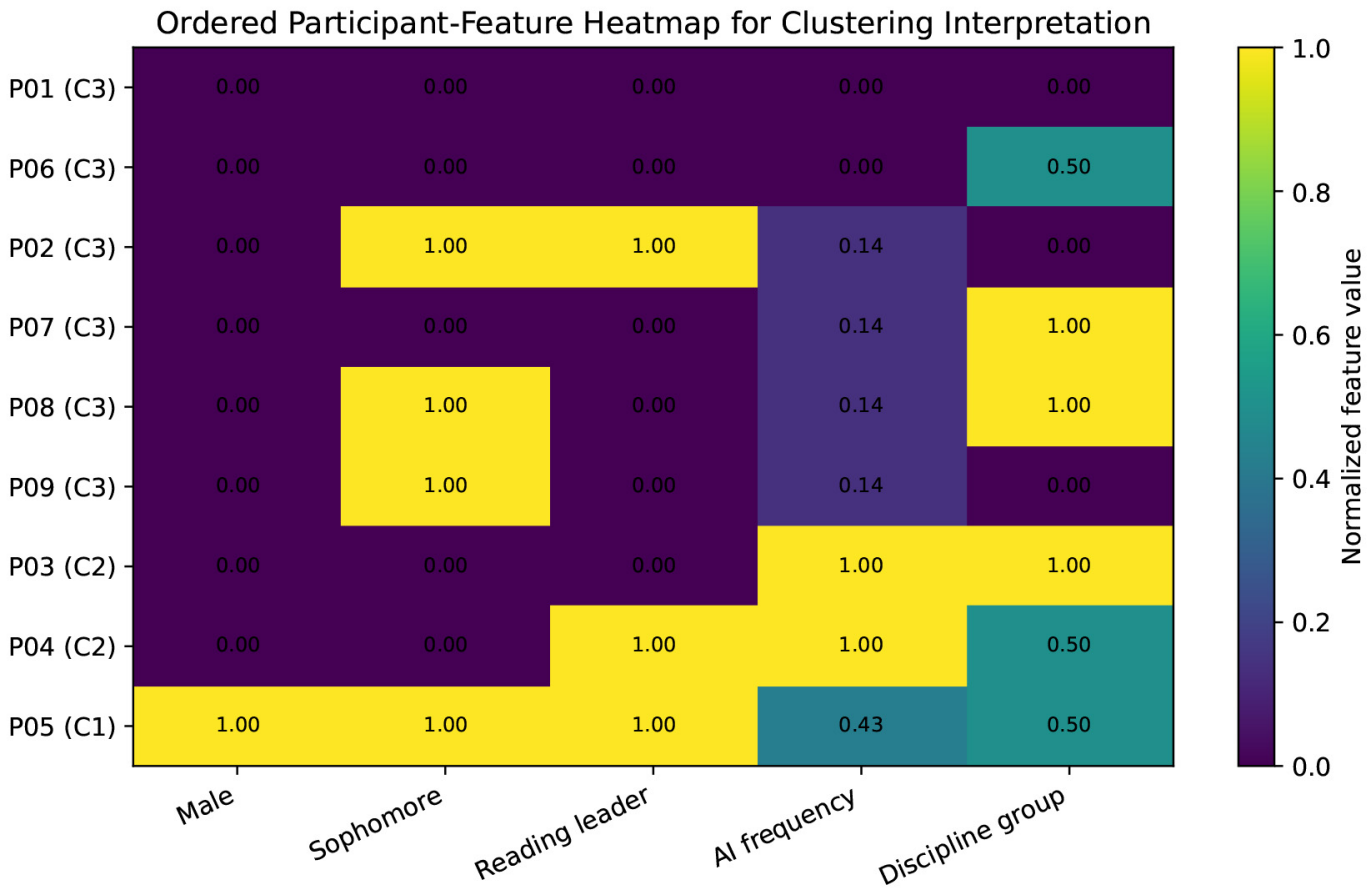
Descriptive participant-feature heatmap; no inferential or predictive interpretation is intended.

The robustness table should be read conservatively. High Cronbach's alpha supports score consistency but may also indicate redundancy; non-significant age and gender tests do not prove equivalence; and the baseline SCL-90 difference remains relevant despite *p* =.059. Most importantly, neither coding agreement nor cross-source convergence removes self-selection or common-method bias.

The PCA, dendrogram, centroid plot, and heatmap are retained only as exploratory visual summaries of nine coded cases. With *n* = 9, component loadings, cluster number, centroids, and apparent separation are highly sensitive to coding choices, scaling, and individual observations; they cannot be validated, generalized, or used to infer stable participant subtypes. The labels “facilitator-engaged,” “high-AI expressive,” and “moderate-use” are descriptive annotations applied to this small sample rather than empirically established classes. No hypothesis test, predictive accuracy, stability resampling, or external validation is claimed. The substantive qualitative interpretation is based on thematic analysis, while these graphics serve only as transparent displays of case-level heterogeneity.

## 6 Conclusion

This study proposed and evaluated a multimodal AI-supported developmental bibliotherapy model in a university library context. By integrating developmental bibliotherapy salons, AI chatbot-supported emotional expression, psychological self-report, participant evaluation, and qualitative interviews, the study examined whether a low-threshold, human-centered, and AI-assisted support model could be feasibly implemented and accepted by university students. Different from purely clinical interventions or algorithm-centered affective computing systems, the proposed model emphasizes the complementary relationship between human-guided reading activities and AI-mediated private emotional disclosure. The university library is therefore positioned not as a substitute for professional counseling services, but as an accessible support environment that can promote self-reflection, emotional expression, interpersonal resonance, and developmental growth.

The empirical findings suggest that the proposed model demonstrated good feasibility and acceptability. Recruitment reached full capacity within two days, the retention rate was 84.4%, and 94.7% of completers reported moderate or considerable benefit. Exploratory quantitative analysis showed a small reduction in SCL-90 scores among intervention participants after the three-week program. Qualitative findings explained this pattern by revealing perceived mechanisms including emotional disclosure, cognitive reframing, interpersonal resonance, and behavioral activation. Additional analyses, including component-level ablation, robustness analysis, error analysis, engagement visualization, and clustering-based profiling, further supported the internal coherence of the proposed model.

In the revised interpretation, the component table is conceptual rather than ablative, and the PCA/clustering figures are purely exploratory visualizations. Neither analysis strengthens causal inference. The most defensible conclusions are that the program could be delivered, most completers evaluated it positively, and participants described distinct perceived functions for group reading and private chatbot interaction.

The study also contributes to the broader topic of multimodal data processing in neuroscience and perception science. This contribution is deliberately narrow: the study does not provide neural, physiological, perceptual-sensor, or computational-fusion evidence. Its primary contribution is a feasibility study of heterogeneous mixed-methods evidence integration in a library-based, AI-supported student wellbeing service. Although this work does not aim to develop a new emotion recognition algorithm, it extends multimodal thinking into a real-world wellbeing-support scenario. The model integrates heterogeneous forms of evidence, including self-reported psychological symptoms, AI-use feedback, participant evaluations, interview narratives, and engagement-profile visualization. This provides an application-oriented example of how multimodal affective and perceptual information can be organized to understand students' wellbeing experiences in context. In particular, the findings suggest that AI-supported systems may be most useful when embedded within human-centered service ecosystems rather than deployed as isolated digital tools.

Several limitations should be acknowledged. First, the intervention was voluntary and non-randomized, so the exploratory psychological changes cannot be interpreted as causal evidence of clinical effectiveness. Second, the study was conducted at a single university, and the sample was relatively small, especially for the qualitative and clustering analyses. Third, AI chatbot use was measured mainly through self-report because backend usage logs were unavailable due to privacy restrictions. Fourth, the intervention lasted only three weeks, so long-term sustainability and delayed effects remain unknown. Future research should use randomized controlled or quasi-experimental designs, recruit larger and more diverse samples, incorporate privacy-preserving AI-use logs, and add longitudinal follow-up.

Additional limitations include incomplete documentation of the AI platform and safety configuration, absence of verified reason-level attrition data in the supplied package, incomplete demographic summary statistics, lack of a reproducible comparison-group follow-up estimate, and absence of source transcripts for quotation verification. These limitations restrict reproducibility and prevent adjustment for academic year, discipline, previous service use, and prior chatbot exposure. A confirmatory study should preregister the allocation and analysis plan, distinguish feasibility from efficacy outcomes, document adverse events, use a complete participant-flow diagram, and follow COREQ for qualitative reporting.

## 7 Ethics, informed consent, risk management, and adverse events

The study flow diagram indicates that informed consent was obtained at baseline. However, the uploaded source package does not contain the name of the approving ethics committee, approval number, approval date, approved protocol, or the exact written-consent language. The final statement should also confirm whether participation was voluntary, whether withdrawal was permitted without penalty, and how participants were informed that the AI chatbot was not a substitute for professional mental-health care.

Because the study involved psychological distress assessment and AI-mediated emotional disclosure. At minimum, it should specify how severe symptoms, suicidality, or acute distress were identified; who reviewed concerning information; which campus counseling, clinical, or emergency services received referrals; and how urgent risk was escalated. The manuscript must also report the verified number of adverse events and concerning chatbot responses. Absence of an event log in the supplied package cannot be interpreted as evidence that zero events occurred.

For privacy protection, it should specify anonymization or pseudonymization, encryption, access permissions, storage duration, deletion procedures, and whether screenshots were redacted before analysis. Platform-provider access to text or voice data, cross-border processing, and reuse for model training should be disclosed where applicable.

## Data Availability

The original contributions presented in the study are included in the article/Ssupplementary material, further inquiries can be directed to the corresponding author.
